# Psychoses and professional activity: Impact on medical fitness for work

**DOI:** 10.1192/j.eurpsy.2023.1011

**Published:** 2023-07-19

**Authors:** A. Belkahla, D. Brahim, H. Ben Said, A. Ghenim, W. Ayed, S. Ernez, I. Youssef, N. Ladhari

**Affiliations:** Occupational pathology and fitness for work, Charle Nicolle Hospital, Tunis, Tunisia

## Abstract

**Introduction:**

Psychoses constitute an extremely heterogeneous clinical entity, with variable medical and socio-professional prognosis depending on several associated factors.

**Objectives:**

- To describe the socio-professional and medical characteristics of patients with psychotic disorders.

- To study the repercussions of these psychotic disorders on the patients’ medical fitness for work.

**Methods:**

Cross-sectional descriptive study of workers with psychotic disorders referred to the consultation of occupational pathology of Charles Nicolle Hospital in Tunis for a medical opinion of fitness during the period from January 2013 to July 2022.

**Results:**

A total of 34 patients were included. The average age was 41.67 ± 10 years. A male predominance was noted with a sex ratio (M/F) of 1.12. Patients with bipolar disorders represented 77% (n=26) of the psychotics versus 23% with schizophrenia (n=8). Two cases had a family history of psychosis. The most represented sector was the health sector in 41% of cases, followed by the tertiary sector in 11.8% of cases. The most prevalent job position was administrative assistant (14.7%). The average professional seniority was 17.07 ± 11.18 years. At the end of the medical aptitude consultation, 17% of the patients (n=6) were considered fit to continue their usual professional activity and 70% of the patients (n=24) had certain restrictions, mainly an exemption from night work in 46% of the cases (n=11) and from security and responsibility functions in 17% of cases (n=4). Temporary unfitness for work was indicated for 18% of patients (n=6) with a median duration of 8 ± 3.46 months. Twenty-three percent (23%) of the patients were judged permanently unfit for their jobs. Early retirement was proposed for five patients. The main diagnosis for permanent unfitness was bipolar disorder (7/8 patients).

**Conclusions:**

The evaluation of the social and professional impact of psychotic disorders is an area of research that requires continuous and periodic re-evaluation.

**Disclosure of Interest:**

None Declared

